# Glucocorticoid receptor and Histone deacetylase 6 mediate the differential effect of dexamethasone during osteogenesis of mesenchymal stromal cells (MSCs)

**DOI:** 10.1038/srep37371

**Published:** 2016-11-30

**Authors:** Marilyn G. Rimando, Hao-Hsiang Wu, Yu-An Liu, Chien-Wei Lee, Shu-Wen Kuo, Yin-Ping Lo, Kuo-Fung Tseng, Yi-Shiuan Liu, Oscar Kuang-Sheng Lee

**Affiliations:** 1 Molecular Medicine Program, Taiwan International Graduate Program, Academia Sinica and Institute of Biochemistry and Molecular Biology, National Yang-Ming University, Taipei 11221, Taiwan; 2 Institute of Biophotonics, National Yang-Ming University, Taipei 11221, Taiwan; 3 Institute of Clinical Medicine, National Yang-Ming University, Taipei 11221, Taiwan; 4 Program in Molecular Medicine, National Yang-Ming University and Academia Sinica, Taipei 11221, Taiwan; 5 Department of Orthopaedics, Cheng-Hsin General Hospital, Taipei 11220, Taiwan; 6 Taipei City Hospital, Taipei 10341, Taiwan; 7 Department of Medical Research, Taipei Veterans General Hospital, Taipei 11217, Taiwan

## Abstract

Lineage commitment and differentiation of mesenchymal stromal cells (MSCs) into osteoblasts *in vitro* is enhanced by a potent synthetic form of glucocorticoid (GC), dexamethasone (Dex). Paradoxically, when used chronically in patients, GCs exert negative effects on bone, a phenomenon known as glucocorticoid-induced osteoporosis in clinical practice. The mechanism on how GC differentially affects bone precursor cells to become mature osteoblasts during osteogenesis remains elusive. In this study, the dose and temporal regulation of Dex on MSC differentiation into osteoblasts were investigated. We found that continuous Dex treatment led to a net reduction of the maturation potential of differentiating osteoblasts. This phenomenon correlated with a decrease in glucocorticoid receptor (GR) expression, hastened degradation, and impaired sub cellular localization. Similarly, Histone Deacetylase 6 *(HDAC6)* expression was found to be regulated by Dex, co-localized with GR and this GR-HDAC6 complex occupied the promoter region of the osteoblast late marker osteocalcin (OCN). Combinatorial inhibition of HDAC6 and GR enhanced *OCN* expression. Together, the cross-talk between the Dex effector molecule GR and the inhibitory molecule HDAC6 provided mechanistic explanation of the bimodal effect of Dex during osteogenic differentiation of MSCs. These findings may provide new directions of research to combat glucocorticoid-induced osteoporosis.

Osteoblasts differentiate from bone- specific lineage committed mesenchymal stromal cells (MSCs)^1^,^2^. Bone lineage commitment is driven by the expression of the transcription factor RUNX2 in MSCs^3^,^4^ which further promotes expression of the early markers Alkaline phosphatase (ALP)^5^, Osterix (OSX), and late markers Collagen type 1 (Col1a1), Osteopontin (OPN), Bone sialoprotein (BSP) and Osteocalcin (OCN). This sequential upregulation leads to osteoblast maturation and deposition of mineralized extracellular matrices^4^. In *in-vitro* culture systems, the differentiation of MSCs into osteoblasts is enhanced by dexamethasone (Dex), a potent synthetic form of the steroid glucocorticoid (GC)^6^,^7^,^8^,^9^. Although it has been widely used to promote osteogenesis^10^, differential effects of Dex on undifferentiated MSCs and osteoblasts were reported^11^,^12^. Specifically, low GC concentration promotes MSCs commitment and enhances differentiation^6^,^8^,^11^,^13^ while high concentrations and long-term treatments inhibit maturation and terminal differentiation of osteoblasts^14^,^15^. This phenomenon has been reasoned to be dependent on the treatment duration, concentration and stage of osteoblast differentiation^7^,^14^. The mechanistic explanation and key mediators of this effect is largely unknown. Several studies have provided explanations on how GCs negatively regulates matured osteoblasts. In animal models for example, excessive GCs were found to suppress genes involved in osteogenesis and mineralization at the later stage^16^, including downregulation and upregulation of positive and negative regulators of osteoblast functions respectively^13^,^17^,^18^. Also, GCs was found to alter the differentiation potential of MSCs by shifting the differentiation away from the osteoblast lineage^19^ suppress proliferation^20^ or inhibit terminal differentiation of osteoblasts^14^,^15^. The underlying mechanisms however of this differential and paradoxic effect of GCs during differentiation of bone-lineage committed MSCs are largely unknown.

The main downstream effector of GCs is the glucocorticoid receptor (GR), a ligand-inducible transcription factor belonging to the nuclear receptor superfamily. In the absence of ligand, GR forms a complex with a multimeric chaperone complex of heat-shock protein 70 (hsp70), hsp90, p23, and immunophilins, among other factors at the cytoplasm. Upon ligand binding, GR dissociates from this complex, translocates into the nucleus and positively regulates transcription by directly binding to specific glucocorticoid response elements (GREs) in the promoter region of its target genes^21^,^22^,^23^. Negative regulation also occurs when GR binds to a negative glucocorticoid response elements (nGREs) having a consensus GRE sequence of 5′ GGTACAnnnTGTTCT 3′ from the transcription start site of the promoter^24^. In osteoblasts, the early bone-specific marker *RUNX2,* the late marker *OCN*^25^,^26^, and *BSP*^27^ are among the direct targets of GR. It was found that GR positively regulates RUNX2 transcription through the direct binding of GR to the *RUNX2* P2 promoter^28^ and inhibits *OCN* through the nGREs on the distal region of the *OCN* promoter^25^,^26^. GR also regulates gene transcription independent of DNA binding through direct protein-protein interactions or facilitates the assembly of other regulatory proteins on target promoters^29^.

Aside from GR regulating transcriptional activity, epigenetic regulation, such as histone acetylation catalyzed by Histone deacetylases (HDACs) has been involved in directing stem cell fate and influences osteoblast differentiation^30^,^31^. HDACs remove acetyl groups in lysine residues of histones and other proteins and alter the chromatin structure, protein stability, protein-protein interactions and recruitment of transcription factors to promoter regions of target genes. Several HDACs contribute to the molecular pathways regulating the specification, maturation and terminal differentiation of osteoblasts^30^,^32^,^33^. For example, the lack of HDAC6 in HDAC6 knock-out mice resulted in a slight increase in cancellous bone mineral density, indicating that it plays a role in bone biology^34^. Furthermore, HDAC inhibition was also found to induce differentiation of stem cells^35^,^36^,^37^. In this study, we present that high dose and long term GC exposure exerted differential effects on differentiating MSCs resulting to a net reduction of maturation potential of osteoblasts. This effect was mediated by a crosstalk between GR and HDAC6 as well as the formation of a GR-HDAC6 repressor complex on the late osteoblast marker Osteocalcin. These findings provided mechanistic explanation of the differential effect of Dex during bone lineage commitment and differentiation of MSCs.

## Results

### Dexamethasone exerted differential effects on the osteogenic differentiation of mesenchymal stromal cells (MSCs)

**Figure 1:**
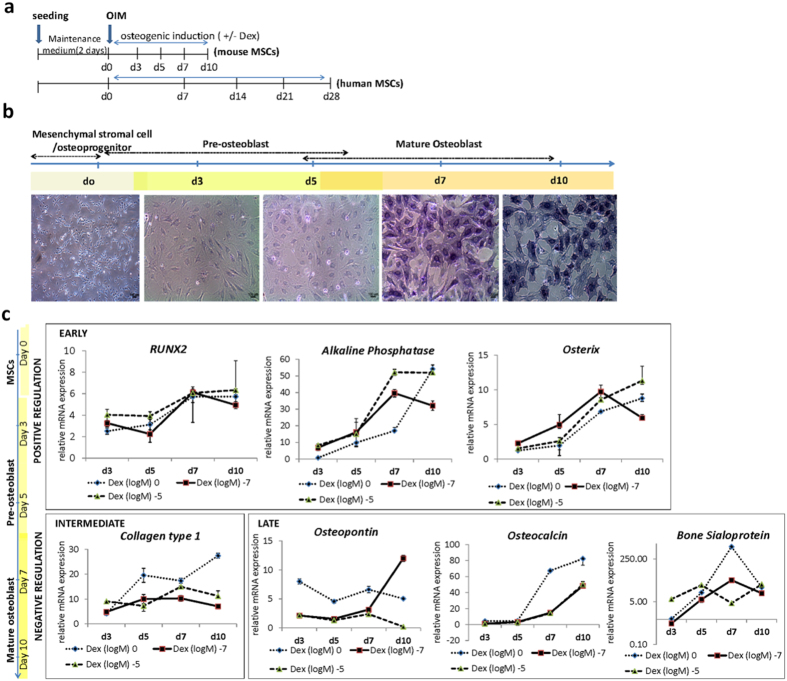
Dexamethasone promotes osteogenic differentiation and exerts differential expression of bone-specific markers in mouse MSCs. (**a**) Schematic representation of osteogenic induction of Mesenchymal stromal cells (MSCs). Undifferentiated MSCs were seeded at an initial density of 1.5 × 10^3^ cells/cm^2^ and maintained in low glucose-DMEM (LG) with 10% serum for 48 hours and replaced with osteogenic induction medium (OIM). Samples were collected at various time points: d0, d3, d5, d7, d10 days (d) for differentiation assays. (**b**) Changes in cellular morphology and Alkaline phosphatase (ALP) activity of mouse MSCs (mMSCs) differentiated into pre-osteoblasts (d3–d5) and osteoblasts (d7–d10) upon osteogenic induction with an optimum Dex concentration of 10^−7^ M. Cells were treated with 5-bromo-4-chloro-3-indolyl phosphate/nitroblue tetrazolium (BCIP/NBT; Sigma) to examine ALP activity. Scale bar = 100 μm. (**c**) Relative mRNA expression by Real time- quantitative PCR (RT-qPCR) of bone specific markers in mMSCs under days (d), 3, 5, 7, 10 of osteogenic induction with three different Dex concentrations: without (0 M), optimum (10^−7^ M) and high (10^−5^ M) Dex. Relative expression was calculated by getting the difference of the *ΔC*_*t*_ of the target gene and RPS18 internal control and under 0 M Dex concentration during osteogenic induction (*ΔΔC*_*t*_). The relative value was expressed as RQ (2^−*ΔΔC*_*t*_). The results are representative of at least three independent trials, each time in triplicate.

The osteogenic differentiation potential of mouse mesenchymal stromal cells (mMSCs) and human mesenchymal stromal cells (hMSCs) were characterized by morphological examination, expression of *ALP* (Fig. 1b, Supplementary Fig. S1, S2) and bone specific markers *RUNX2, OSX, COL1, OCN, OPN* and *BSP* (Fig. 1c).

**Figure 2:**
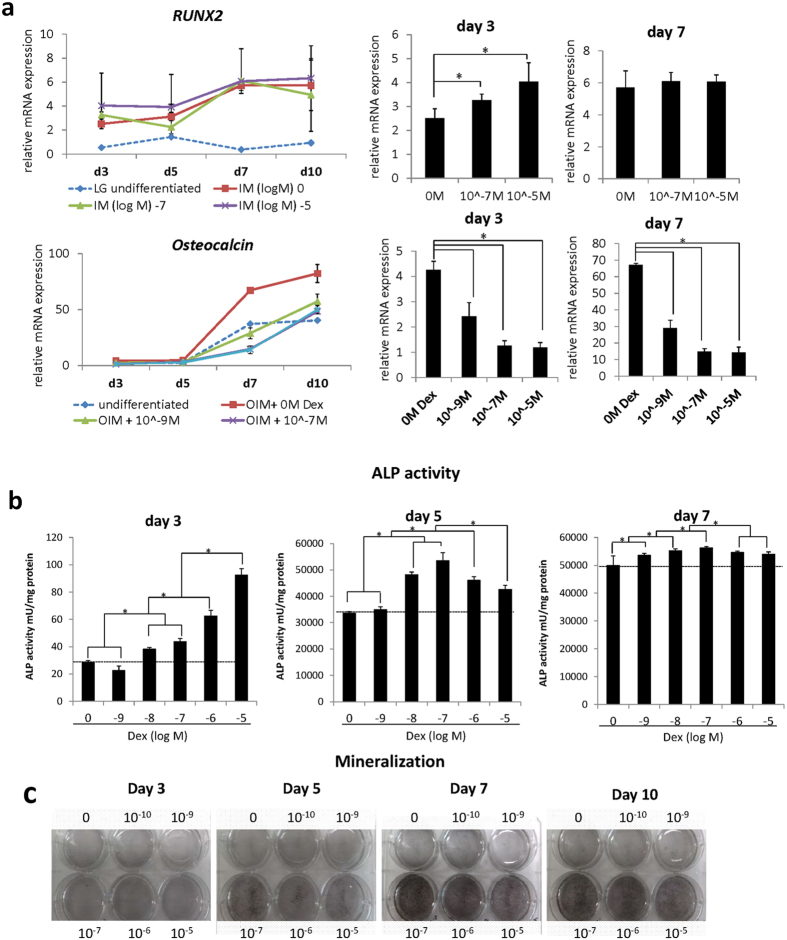
Dexamethasone enhances osteogenesis at the early stage however decreases mineralization at a later stage. (**a**) Gene expression of the early marker of bone differentiation (*RUNX2)* and the late marker *Osteocalcin (OCN)* was determined by Real time- quantitative PCR (RT-qPCR) during osteogenic induction with 0 M, 10^−7^ M and 10^−5^ M Dex. Gene expression of mMSCs in maintenance medium (Low Glucose DMEM; LG) was also examined for spontaneous differentiation. Data were represented as mean ± SD. Asterisks indicate a significant difference p < 0.05 (N = 3). (**b**) Alkaline phosphatase activity (ALP) activity (early stage) of mMSCs under different Dex concentrations (10^−9^ to 10^−5^ M). ALP activity was normalized to protein content of the cell lysate (ALP activity/mg protein). Asterisks indicate a significant difference p < 0.05 (N = 5). (**c**) Von kossa staining to assess mineralization (late stage) in mMSCs beginning at day 3 of osteogenic induction under 5 different Dex concentrations (10^−9^ to 10^−5^ M). At least three independent repeats were performed, each time in triplicate.

To examine the effect of high-dose and long-term treatment of Dex during osteogenesis, mMSCs were cultured in osteogenic induction medium (OIM) for 10 days under three conditions: no Dex treatment (0 M), medium Dex concentration of 10^−7^ M, and high dose of 10^−5^ M (Fig. 1a). Results of Reverse Transcription - Quantitative Polymerase Chain Reaction (RT-qPCR) demonstrated significant dose-dependent increase of the early marker *RUNX2*, and its known transcriptional targets *ALP* and *OSX* as early as day 3 (Figs 1c and 2a). Long-term treatment however, diminished this dose-dependent effect, and *Runx2* expression was unresponsive to Dex treatment (Figs 1c and 2a). Increasing concentration of Dex however, did not affect the protein level of RUNX2 during osteogenic differentiation (Supplementary Fig. S3). As differentiation progresses however, the presence of Dex significantly reduced the expression of the late markers *Col1a1, OPN, OCN* and *BSP* as compared to the no Dex treatment group. Interestingly, a sustained inhibitory effect was observed on *OCN* as early as day 5 (Figs 1c and 2a) and the presence of Dex alone rendered *OCN* expression inhibitory regardless of the concentration (Fig. 1c).

ALP activity (early stage) and mineralization (late stage) were further assessed. Figure 2b showed that ALP activity peaked at day 5 compared to control. During the later stage and at high concentration however, no significant difference on ALP activity was observed from all treatment groups (Fig. 2b). On the other hand, Von Kossa staining demonstrated a peak of mineralization at day 7, however prolonged Dex treatment at high concentration inhibited mineralized matrix production at day 10 (Fig. 2c).

These results indicated that dexamethasone exerted both positive and negative effects (differential) on bone-specific markers during MSCs osteogenic induction and rendered a net inhibitory effect on the maturation of differentiating osteoblasts.

### High concentration of Dexamethasone altered glucocorticoid receptor (GR) sub-cellular localization and hastened GR degradation

Since we found a dose dependent positive induction by Dex on osteoblast genes particularly *RUNX2* during the early stage, and a diminished effect during long-term Dex treatment, we further examined whether the main effector molecule, glucocorticoid receptor (GR) similarly shows differential responses during differentiation. Immunofluorescence staining showed GR expression in mMSCs, hMSCs, and mature osteoblast cell line human fetal osteoblast (hFOB) (Supplementary Fig. S4). Expectedly, GR was localized in the cytoplasm in the absence of ligand and translocated into the nucleus upon Dex treatment (Supplementary Fig. S5). Interestingly, high concentration of Dex (10^−5^ M) caused a retention of the receptor complex in the cytoplasm as compared to 10^−7^ M (Supplementary Fig. S5, S6, S7), suggesting a delay or blockade of cytoplasm-nucleus trafficking of the GR-ligand complex.

**Figure 3:**
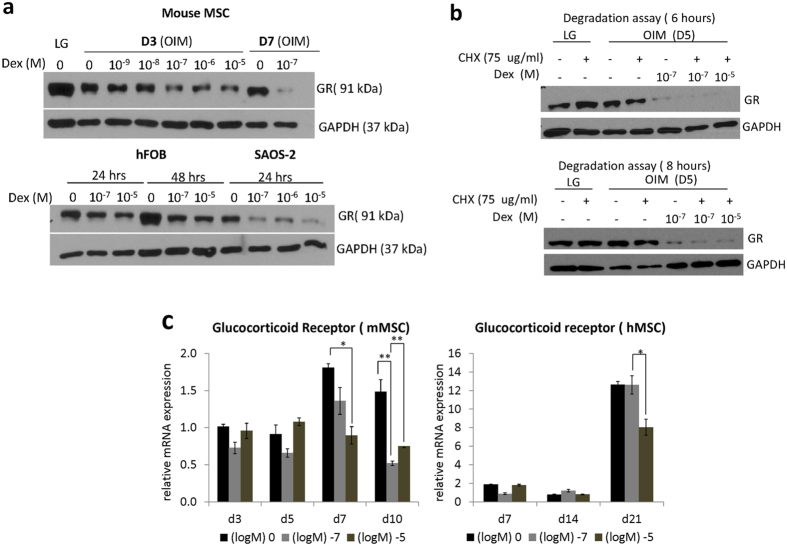
Continuous and high concentration of dexamethasone hastened glucocorticoid receptor (GR) degradation. (**a**) Protein expression of GR was detected from MSCs under maintenance medium (LG) and osteogenic induction medium (OIM) for 3 and 7 days (D3, D7) with 0 M, 10^−9^ to 10^−5^ M Dex and from mature bone cells: Human Fetal Osteoblast (hFOB) and osteosarcoma cell line (SaOS-2) seeded at a density of 2.5 × 10^5^ *cells*/T25 flask, maintained in IMDM and treated with 0 M, 10^−7^ M and 10^−5^ M Dex for 24 and 48 hours. Samples were immunoblotted using total GR antibody and GAPDH as loading control. Full-length blots are presented in Supplementary Fig. S12 (**b**) Cyclohexamide (CHX) treatment in undifferentiated mMSCs maintained in low glucose (LG) DMEM and differentiating MSCs at day 5 in OIM. De-novo GR protein synthesis was inhibited by 75 μg/ml cyclohexamide (CHX) for 6 hours and 8 hours treated in the presence or absence of 10^−7^ and 10^−5^ M Dex. Samples were immunoblotted against total GR antibody and GAPDH as loading control. (**c**) MSCs were cultured under Osteogenic Induction Medium (OIM) at days 3, 5, 7, 10 days in mouse MSCs and 7, 14, 21 in human MSCs and relative GR mRNA expression was determined by Real time- quantitative PCR (RT-qPCR). Results were represented as mean ± SD (N = 3). Asterisks indicate a significant difference p < 0.05.

To examine the effect of Dex on GR protein expression, immunoblotting was performed and showed a time and dose dependent decrease of GR in mMSCs and hMSCS, as well as in osteoblast cell lines hFOB (Fig. 3a, Supplementary Fig. S6, S12). Furthermore, treatment of mMSCs with cyclohexamide (Fig. 3b, Supplementary Fig. S12) at day 5 of osteogenic differentiation validated the hastened degradation of GR upon high dose and long term Dex treatment and could have accounted to the loss of inductive response and downregulation of *RUNX2, ALP* and *Osterix* during the later stage of differentiation (Fig. 1c and 2a). In addition, high dose of Dex also caused a significant decrease in mRNA level of total GR during the later stages (day 7–10 in mMSCs and day 21 in hMSCs) (Fig. 3c).

**Figure 4:**
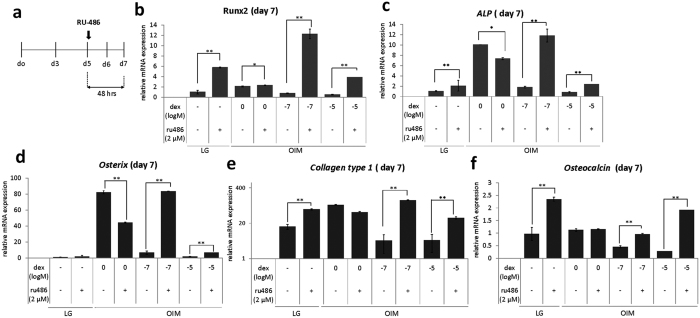
GR inhibition by RU-486 promotes osteogenic differentiation at the later stage. (**a**) Schematic representation of RU-486 treatment (2 μM) for 48 hours in mMSCs at day 5 of osteogenic induction. (**b–f**) Relative bone-specific gene expression of mMSCs by Real time- quantitative PCR (RT-qPCR) upon treatment of 2 μM RU-486 for 48 hours and harvested at day 7 of osteogenic induction. The results are representative of at least two independent trials, each time in triplicate (N = 3). *p < 0.05; **p < 0.01.

Previous reports have demonstrated GR autoregulation^38^,^39^ and we speculated that GR downregulation was caused by this negative feedback effect. To test whether the decrease of inductive response of *RUNX2, ALP, and Osterix* (Figs 1c and 2a) at the late stage of osteogenesis was mediated by GR during continuous Dex treatment, we treated differentiating osteoprogenitors with GR antagonist RU-486 at day 5, since this period appeared to be the time window/stage when early osteogenic markers ceased to respond to Dex treatment (Fig. 1c) and the time point of onset of GR degradation (Fig. 3a). Results showed that after 48 hours of the treatment, a significant upregulation of *RUNX2, ALP, Osterix* and *COL1* was observed (Fig. 4). In addition, the late marker of osteoblast maturation, *OCN*, was also upregulated in the presence of RU-486 (Fig. 4).

These results indicate that GR protein stability and cytoplasm-nucleus trafficking accounts to the differential effect of Dex during the early and late stages of osteogenesis.

### HDAC6 was induced by dexamethasone and co-localized with GR during osteogenic differentiation

**Figure 5:**
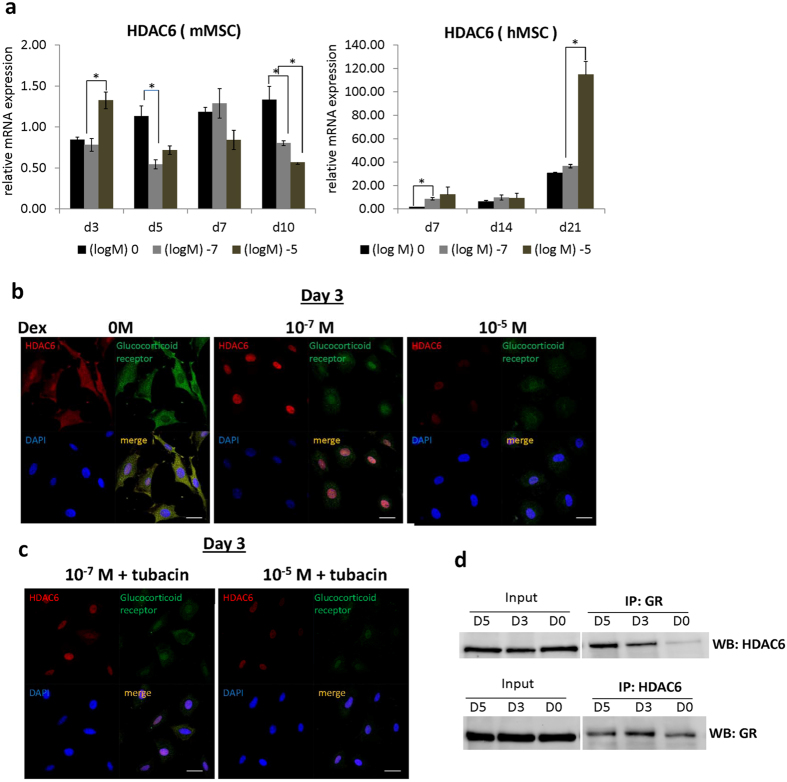
Dexamethasone regulates Histone Deacetylase 6 (HDAC6) through the glucocorticoid receptor (GR) in differentiating MSCs. (**a**) Relative mRNA expression of HDAC6 by RT-qPCR in differentiating mouse MSCs (mMSCs), human MSCs (hMSCs) and terminally differentiated human fetal osteoblasts (hFOB) under treatment with 0 M, 10^−7^ M and 10^−5^ M Dex concentrations. Results are representative of at least two independent repeats. Asterisks indicate a significant difference; (N = 3) *p < 0.05; **p < 0.01. (**b**) Cytoplasmic and nuclear localization of GR and HDAC6 in MSCs at day 3 of osteogenic induction with 0 M, 10^−7^ M and 10^−5^ M Dex. Cells were stained with total GR and HDAC6 with Alexa Fluor 488-conjugated goat anti-rabbit and Alexa Fluor 586-conjugated goat anti-mouse secondary antibodies respectively, counterstained with DAPI, imaged under Zeiss LSM 700 confocal microscope and processed using LSM Image Browser (Zeiss). Scale bar = 20 μm. (**c**) HDAC6 inhibition by 2 μM Tubacin for 24 hours in mouse MSCs (mMSCs) at day 3 of osteogenic induction with 10^−7^ M Dex. (**d**) Complex formation between GR and HDAC6 by Co-immunoprecipitation from mouse MSCs at days 0, 3, 5 (D0, D3, D5) of osteogenic induction with 10^−7^ M Dex. Full-length blots are presented in Supplementary Fig. S13 IP: immunoprecipitation; IB: immunoblot.

We next sought to identify a putative regulatory molecule induced by Dex through GR which accounts to the decreased maturation potential during the late stage of osteogenesis. RT-qPCR revealed the expression of *HDAC6* in mMSCs and in committed osteoprogenitors as early as day 3 of differentiation. High Dex concentration (10^−5^ M) significantly enhanced gene expression of *HDAC6* at day 3 in mMSCs of osteogenic induction and at day 7 in hMSCs (Fig. 5a). Interestingly, a decrease in *HDAC6* mRNA level was observed at day 10 in mMSC (Fig. 5a), concomitant to a decrease in GR protein level during long term Dex treatment (Fig. 3b), suggesting that *HDAC6* was slightly responsive to the differential/bimodal Dex regulation. We further explored this by determining the protein expression and localization of endogenous HDAC6 during osteogenesis. Immunofluorescence staining showed protein expression of HDAC6 in mMSCs at day 3 and day 7 of osteogenic induction (Fig. 5b, Supplementary Fig. S8). HDAC6 was predominantly found in the cytoplasm, distinct from other nucleus-localized HDAC types. In the presence of Dex, HDAC6 similarly translocated to the nucleus similar to GR. Figure 5b also demonstrated the complex formation between HDAC6 and GR at day 3 of osteogenic induction. Co-Immunoprecipitation assay further confirmed this GR-HDAC6 complex formation at day 3 and day 5 of osteogenic differentiation (Fig. 5d, Supplementary Fig. S13). GR co-localized with HDAC6 even in the absence of the ligand, although intense co-localization puncta between HDAC6 and GR appeared in both cytoplasm and nucleus in the presence of Dex (10^−7^ M). Although it up-regulated mRNA level at day 3 of osteogenic induction (Fig. 5a), high concentration of Dex (10^−5^ M) however, decreased HDAC6 protein expression (Fig. 5b). At high Dex concentration, the GR-HDAC6 complex weakened though not completely abolished which could either be explained by partial degradation of GR at prolonged and high Dex concentration or a decreased in HDAC6 protein expression.

To further determine whether this response of HDAC6 to Dex is mediated by GR, mMSCs under 10^−7^ M osteogenic induction were treated with RU-486 for 24 hours and showed a decrease HDAC6 expression (Supplementary Fig. S10) confirming the role of GR in regulating HDAC6 upon Dex treatment. In addition, treatment with an HDAC6 specific inhibitor Tubacin reduced the complex formation between GR and HDAC6 (Fig. 5c).

Taken together, the result suggested a potential co-regulatory function of the GR-HDAC complex during osteogenic differentiation that could influence osteoblast maturation during the later stages of osteogenesis.

### Dexamethasone recruited the GR-HDAC6 complex to the negative regulatory element of the osteocalcin (OCN) promoter

**Figure 6:**
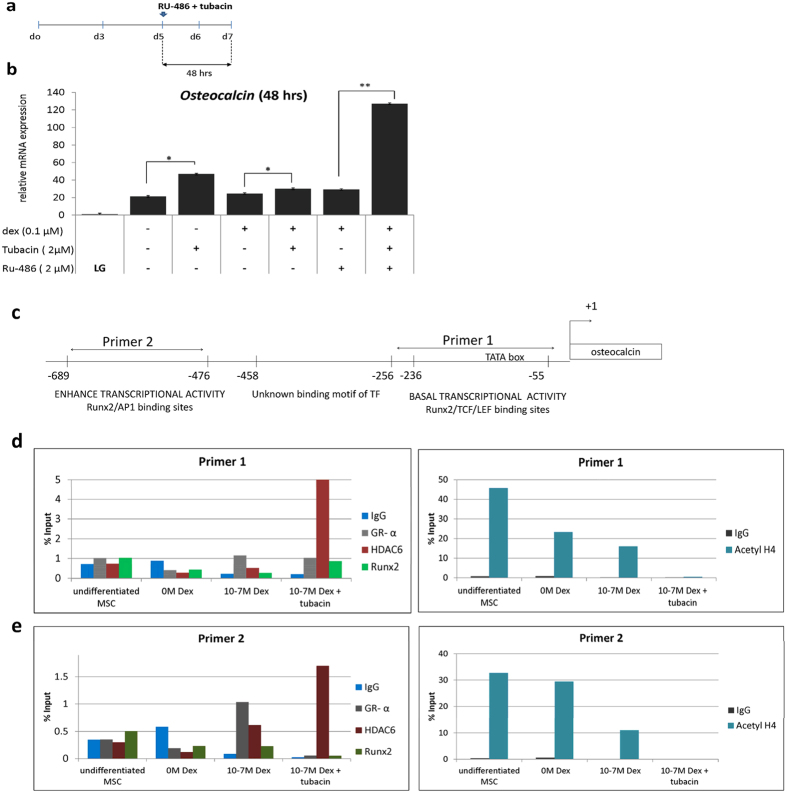
GR-HDAC6 complex binds to the promoter region of *osteocalcin (OCN)* and regulates its expression. (**a**) Schematic representation of combinatorial inhibition of GR by RU-486 (2 μM) and HDAC6 by Tubacin (2 μM) for 48 hours in mouse MSCs (mMSCs) at day 5 of osteogenic induction with 10^−7^ M Dex. Samples were collected after 48 hours (day 7 of differentiation) (**b**) *OCN e*xpression was analyzed by RT-qPCR using RPS18 as internal control upon combinatorial inhibition by RU-486 and Tubacin. LG (low glucose)-maintenance medium of undifferentiated MSCs; OIM (osteogenic induction medium) of differentiating MSCs. Results are representative of two independent trials, each time in triplicate. Asterisks indicate a significant difference; (N = 3) *p < 0.05; **p < 0.01. (**c**) Schematic representation of the proximal region (Primer 1) and distal region (Primer 2) of the OCN promoter. The occupancy of GR, HDAC6 and Runx2 on the proximal (**d**) and distal regions (**e**) of the OCN promoter was examined by chromatin immunoprecipitation in undifferentiated and differentiating mMSCs (10^−7^ M Dex) with and without Tubacin at day 5 of osteogenic differentiation. Samples were harvested after 24 hours (day 6 of osteogenic differentiation). Acetyl-H4 antibody was used to verify acetylation status of groups upon inhibitor treatment. 3′ UTR was used as a negative control. The ChIP-RT-qPCR data was normalized with percent Input.

Based on our findings that GR inhibition by RU-486 up-regulated *OCN* (Fig. 4f), GR-HDAC6 forming a complex (Fig. 5b, Supplementary Fig. S8) and both OCN, GR, HDAC6 were responsive to continuous Dex treatments (Figs 4 and 5b), we further investigated the role of HDAC6 on the expression *OCN*, a late marker of osteoblast. Differentiating osteoblasts at day 7 of cultures were treated with Tubacin, a specific HDAC6 inhibitor, and the level of mRNA of *OCN* transcript was analyzed. As expected, Dex treatment inhibited *OCN* mRNA level and upon inhibition of HDAC6 by Tubacin, *OCN* expression was enhanced (Fig. 6b) suggesting that HDAC6 contributes to the negative regulation of *OCN* during osteogenesis in the presence of dexamethasone.

To further investigate whether the HDAC6-GR complex plays a role on the repression of *OCN* transcription during long term Dex treatment, combinatorial treatment of RU-486 and Tubacin was applied to the culture at day 5 of osteogenic induction. Results showed that the relative mRNA level of *OCN* increased 6-folds after 48 hours of combinatorial treatment with the GR antagonist RU-486 and specific HDAC6 inhibitor Tubacin (Fig. 6a). This clearly indicated that the GR-HDAC6 complex acted as a transcriptional repressor of *OCN* during long term treatment of Dex in differentiating osteoblasts.

To identify the mechanism of Dex mediated repression on OCN by the HDAC6-GR complex, chromatin immunoprecipitation was performed to determine the occupancy of this complex to the *OCN* promoter in mMSCs cultures at day 5 of induction. Although it is known that the *OCN* promoter has a negative glucocorticoid response elements (GREs)^40^, we hypothesized that HDAC6 forms a co-repressor complex with GR to inhibit *OCN* expression.

Three primer pairs were used for the CHIP assay (Fig. 6c, Supplementary Fig. S9) with primer 1 targeting the proximal region of the OCN promoter responsible for OCN basal tissue specific transcription and containing a Runx2 binding site^41^, primer 2 for the distal region known for enhanced OCN transcriptional activity^33^ and primer 3 targeting a 3′ UTR primer as a negative control.

Results showed the occupancy of the GR receptor at both proximal (155 to −236) and distal regions (155 to −236) of the OCN promoter upon Dex treatment (10^−7^ M), in contrast to the undifferentiated and no Dex treatment group relative to the IgG controls (Fig. 6d). To validate the potency of the inhibitor, the acetyl H4 status in all groups were determined. As expected, the acetylation status of the osteocalcin promoter decreased in the presence of Tubacin. Interestingly, in the presence of the inhibitor, the enrichment of GR at the proximal region and more so at the distal region decreased (Fig. 6d). On the other hand, RUNX2 showed increased occupancy at the −55 to −236 (primer 1) site of the osteocalcin promoter (Fig. 6d) suggesting displacement of GR and recruitment of RUNX2 in the presence of the inhibitor. Unexpectedly, HDAC6 was still bound to the OCN promoter even in the presence of Tubacin (Fig. 6d,e), suggesting that HDAC6 might be playing a role other than modifying the histone acetylation status of the OCN promoter.

Taken together, a GR-HDAC6 repressor complex existed at both the proximal and distal region of the osteocalcin promoter. Tubacin treatment reduced the occupancy of GR to nGRE of the OCN promoter, and was associated with the enhancement of *OCN* expression during the later stage of osteogenic induction.

## Discussion

**Figure 7:**
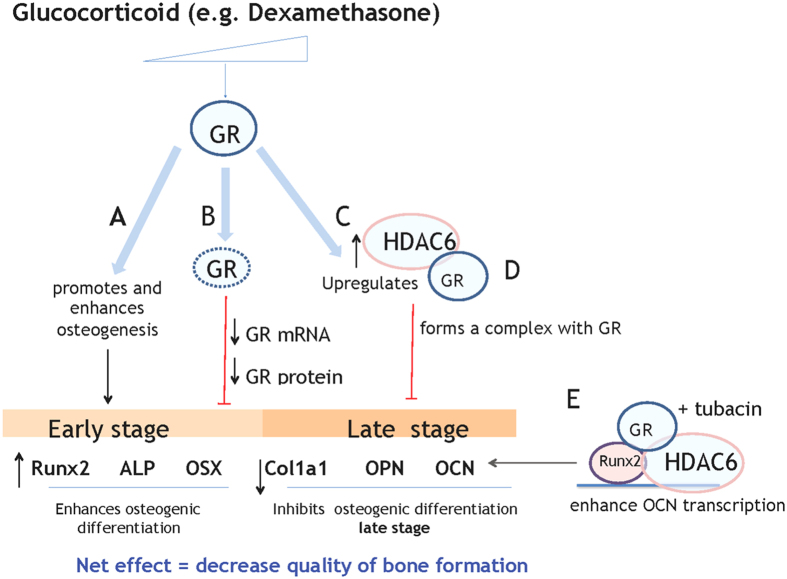
Mechanism of the role of GR-HDAC6 complex on the differential effect of Dex during osteogenic differentiation of Mesenchymal stromal cells (MSCs) was proposed. (**A**) At the early stage of osteoblast commitment and differentiation, dexamethasone (Dex) promotes bone-lineage commitment of MSCs and enhances the expression of *e*arly osteoblast marker genes *Runx2, Alkaline phosphatase (ALP*) and *Osterix (OSX*) through the effector molecule Glucocorticoid receptor (GR). (**B**) Upon long term and high Dex treatment, GR protein expression decreases in a time and dose dependent manner (as early as pre-osteoblast stage; day 5 in mouse MSC) leading to a decrease or unresponsive induction of *RUNX2, ALP* and *OSX* to Dex. (**C**) Dex also induces the expression of an inhibitory protein Histone Deacetylase 6 (HDAC6) and forms a complex with GR. (**D**) The binding of this complex on the promoter region of the late marker *Osteocalcin (OCN*) decreases mineralization at the later stage. (**E**) The treatment of Tubacin during the late stage decreased occupancy of GR to the negative response element of OCN, either by further degradation or displacement of GR by HDAC6, and recruited RUNX2 to the HDAC6-GR complex which may switch as an activator to enhance *OCN* expression and enhance mineralized matrix production.

Glucocorticoids are widely used in immunosuppressive therapies however prolonged use causes a rapid decline of bone mineral density attributed to an imbalance between bone formation and resorption^42^,^43^. Paradoxically, GCs have been utilized *in-vitro* to promote osteogenesis from MSCs^6^,^7^,^8^,^9^,^44^. In the present study, we aimed to examine the temporal and dose dependent regulation of Dex during differentiation, and identified the mechanism on how Dex exerts this differential/bimodal effect (Fig. 7). We have identified the glucocorticoid receptor (GR) and its co-repressor complex formation with HDAC6 as mediators of this effect and provided insights on how GCs rendered a net inhibitory outcome during long term GC treatment.

We validated that GC indeed exert differential effects during osteogenesis leading to reduced turnover of matured osteoblasts. At the early stage of differentiation, the enhancement of *RUNX2, ALP* and *OSX* expressions by Dex was observed and conforms with previous findings^7^,^8^,^45^. *RUNX2* transcription is positively regulated by Dex through the direct binding of the activated Dex-GR complex to the *Runx2* P2 promoter^28^ and consequently driving the expression of *ALP, OSX* and other late marker genes^8^,^46^. GR protein expression however decreased during long term Dex treatment and thus it is putative to reason that the negative effect of Dex at the later stage is caused by a reduced/loss of GR signaling. This decrease of protein turnover upon Dex treatment is attributed to either GR degradation via the ubiquitin-proteasomal pathway^47^, a rapid decrease of GR primary transcript following Dex treatment^48^ or a negative feedback loop or auto regulation occurring in GR^38^,^39^ due to the presence of a functional negative glucocorticoid response element (nGRE) in exon 6 of the GR gene. In MSCs, we observed that the onset of this degradation appears at day 5 of osteogenic differentiation and we used this time window to inhibit GR activity by RU-486 to prevent this feedback loop leading to degradation. Interestingly, RU-486 treatment at this time window significantly upregulated *RUNX2, ALP, Osterix* and *Collagen.* This confirms that the effect on osteoblast specific gene regulation is mediated by GR and provides insights that the stage of osteogenic differentiation in which GR is highly stable and moderately expressed, is a critical factor to target enhancement of maturation potential during prolonged Dex exposure. Furthermore, we also found that sub cellular localization of the GR is equally important in determining the effect of Dex during MSC differentiation and we found that high Dex concentration caused retention of GR in the cytoplasm as compared to 10^−7^ M suggesting a delay or blockade of cytoplasm-nucleus trafficking of the Dex-GR complex. We are currently investigating the role of the cytoskeleton on this phenomenon. Indeed, the protein turnover, stability and localization of GR is critical and explains the differential effect of Dex during the early and late stages of differentiation.

GR also regulates gene transcription independent of DNA binding either through direct protein-protein interactions^49^ or facilitating the assembly of other regulatory proteins on the promoter regions of its target genes^22^. We further speculate that long term treatment of Dex induced an inhibitory protein that forms a complex with GR and changes the chromatin conformation of osteoblast genes. In this study, we found that Histone Deacetylase 6 (HDAC6) is expressed in MSCs, is under regulation by Dex as evident by an increase of mRNA level and a decrease in protein expression during high and long term Dex treatment similar to GR. Previous studies have demonstrated the role of HDAC6 in regulating GR, such that HDAC6 can cause deacetylation of the heat shock protein 90 (Hsp90), crucial for GR chaperone activity, ligand binding, translocation and gene activation^23^,^50^,^51^. The loss of GR signaling during the early-late transition of differentiation may also be explained by Dex-induced HDAC6 expression leading to reduced expression of early osteoblast genes subsequently decreasing matured osteoblast turnover. Intriguingly, long term and high Dex concentration also decreased HDAC6 expression similar to a decrease of GR expression during the later stage of osteogenic induction. This further leads us to consider that a cross talk exists between GR and HDAC6 and both regulates each other during osteogenic induction. It is yet to be explored whether HDAC6 has a GR regulatory element on it promoter and other mechanisms on how Dex directly or indirectly regulates HDAC6 through GR. Nevertheless, we identified that this differential effect of Dex on differentiating osteoblasts is caused by a GR-HDAC6 complex. Furthermore, we also observed colocalization of HDAC6 with GR. It was identified that a LXXLL 313–418 motif in HDAC6 is in contact with the GR-Dex complex in breast cancer T47D and HeLa-GR cells^52^, to our knowledge, this is the first report of a GR-HDAC6 complex existing in MSCs during osteogenic differentiation.

We further identified the exact role of HDAC6 and the HDAC6-GR complex in influencing the maturation potential of differentiating osteoblasts by determining the expression of *Osteocalcin (OCN*). *OCN* is considered a late marker of osteogenic differentiation and its expression at high levels indicates maturation and terminal differentiation of osteoblasts^53^. Although Dex treatment inhibited *OCN* expression due to the direct binding of Dex-activated GR to the nGRE of the osteocalcin promoter^40^, our results showed that HDAC6 is part of a GR complex on this region. Upon adding a specific HDAC6 inhibitor Tubacin during osteogenic induction, we observed an enhanced gene expression of *OCN* compared to the untreated group. In addition, combinatorial treatment with both Tubacin and RU-486 showed almost 6-folds increase of *OCN* mRNA expression. This suggests the role of a GR-HDAC6 complex acting as a transcriptional repressor of *OCN* during long term treatment of Dex in differentiating osteoblasts. Also, upon tubacin treatment, a decreased enrichment of GR at the proximal region was observed which possibly have lifted the repressive effect of GR bound to the nGRE of OCN. Intriguingly, HDAC6 was still found to be bound to the OCN promoter in the presence of the inhibitor. Tubacin targets only one of the two catalytic domains of HDAC6^54^, whether this catalytic domain has structural connections with GR is still unknown. However, we hypothesized that the presence of Tubacin have altered protein-protein interaction or possibly recruited another molecule for competitive binding relieving this repressive effect of GR on OCN thus driving OCN gene expression. We speculated that this molecule is RUNX2 since it has been reported to interact with HDAC6^55^, a physical interaction exists between RUNX2 and the GC receptor (GR)^56^ and the proximal and distal regions of the *OCN* promoter has a Runx2 and TCF/LEF binding sites, and is responsible for basal tissue specific^41^ and enhanced transcriptional transcription^33^ respectively. Furthermore, RUNX2 is known to either act as an activator or repressor depending on the regulatory proteins bound to it^57^. Conversely, the presence of HDAC6 inhibitor could have caused structural changes to recruit RUNX2 to the HDAC6-GR complex and displace GR thus attenuating the repressive effect of Dex on *OCN*. Further studies on the direct regulation of GR on HDAC6 and the involvement of structural conformational changes occurring between the GR-HDAC6-RUNX2 complexes may further be investigated.

These findings may provide insights on modulating the activity of mesenchymal stem cells during glucocorticoid exposures to positively regulate or minimize the effect of GCs during bone formation. In bone formation, the bone marrow provides a continuous supply of bone precursor Mesenchymal stromal cells that will proliferate, differentiate into osteoblasts and synthesize components of a hardened mineralized matrix characteristic of bones. It is essential that the population and quality of these reservoir cells with osteogenic potential be maintained during bone remodeling and healing of bone fractures. In glucocorticoid induced osteoporosis however, continuous glucocorticoid exposure has led to impaired proliferation, a shift away from osteoblast lineage potential and inhibition of osteoblast activity. It cannot be disclaimed however that glucocorticoids render positive effects *in-vitro* studies as it showed enhancement of osteoprogenitor differentiation from lineage committed cells. This study provided insights on how to yield more osteoblast-lineage committed MSCs at the same time promote terminally differentiated osteoblast population during osteogenesis in the presence of glucocorticoids. By allowing ligand-bound GR to positively stimulate osteoblast lineage commitment and differentiation in the presence of dexamethasone in the early stage, at the same time inhibiting GR autoregulation and activity of the ligand-induced inhibitory molecule HDAC6 by inhibitors or antagonist treatment as differentiation progresses, bone formation might be enhanced while minimizing the negative adverse effects of continuous glucocorticoid exposure.

Our findings put forward a proposed two-step protocol *in-vitro*, involving the addition of Dex during the early stage to enhance lineage commitment by enhancing *Runx2, Alp* and *Osx* expression and withdrawal at the later stages or alternatively inhibiting HDAC6 activity by Tubacin or combinatorial treatment of Tuabcin and the GR antagonist RU-486 as early as day 5 (in mouse) and day 14 (in human) to potentially yield more osteogenic lineage committed MSCs and differentiated osteoblasts.

In conclusion, the mechanistic insights on how dexamethasone alters the differentiation fate of osteoprogenitors by decreasing osteoblast specific gene expression and mineralization at the later stage upon long-term GC exposure have been provided. These results suggest that by modulating combinatorial treatment of glucocorticoid with GR and HDAC6 inhibitors or antagonists, the turnover of mature osteoblast is enhanced and may promote a more favorable response against side effects in patients with long-term GC treatment.

## Methods

### Isolation and expansion of mouse and human MSCs

Mouse bone marrow-derived MSCs (mMSCs) were obtained from the femoral and tibial bone marrow of 7–8 weeks old Balb/c mice as previously reported [60]. The protocols were approved by the Taipei Veterans General Hospital Institutional Animal Care and Use Committee (IACUC). All studies involving animals were in accordance with appropriate guidelines. mMSCs were maintained in Low-Glucose DMEM (LG-DMEM; Invitrogen) with 10% fetal bovine serum, and 1% PSG. Commercially available human MSCs (hMSCs) (Steminent Biotherapeutics Inc, Taipei, Taiwan) were maintained in Iscove’s modified Dulbecco’s medium (IMDM; Sigma-Aldrich), supplemented with 10% fetal bovine serum, 10 ng/ml fibroblast growth factor, 10 ng/ml epidermal growth factor (R&D systems, Inc.) and 100 U Penicillin-1000 U Streptomycin-2 mM L-glutamine (1% PSG; Sigma-Aldrich).

### Osteogenic differentiation of MSCs and other osteoblast cell lines

For osteogenic induction of mMSCs, cells were seeded at a density of 1.5 × 10^3^ cells/cm^2^ and maintained in LG-DMEM with 10% serum for 48 hrs, and changed to osteogenic medium (OIM) consisting of HG-DMEM supplemented with 0.2 mM ascorbic acid and 10 mM β-glycerol phosphate with varying concentrations of dexamethasone (Sigma-Aldrich) from 10^−9^ M to 10^−5^ M. For hMSCs, cells were seeded at a density of 4 × 10^3^ and maintained in IMDM (Invitrogen) containing 10% fetal bovine serum, 1% PSG, 10 ng/ml basic fibroblast growth factor (FGF2) (Sigma-Aldrich), and 10 ng/mL epidermal growth factor (EGF) for 24 hours and changed to IMDM-based osteogenic medium (OIM). The medium was replaced every 3 days.

Human Fetal Osteoblast (hFOB) and osteosarcoma cell line (SaOS-2) were purchased from Sigma-Aldrich (St. Louis, MO, USA), seeded at a density of 2.5 × 10^5^ *cells*/T25 flask, and maintained in IMDM supplemented with 10% fetal bovine serum and 1x PSG.

### RNA Extraction and Real Time Reverse Transcription Polymerase Chain Reaction

Total RNA was isolated using Trizol reagent (Invitrogen) and reversed transcribed using MMLV Reverse Transcriptase 1st-Strand cDNA synthesis Kit (EPICENTRE^®^ Biotechnologies). Quantitative real-time PCR (qPCR) was performed with TaqMan® Fast Universal PCR Master Mix (2X) by Step One plus real-time PCR system (Applied Biosystems) to determine the relative gene expression profiles. The detailed protocol can be found in Supplementary Information. The primers used for qPCR are listed in Supplementary Table S1.

Relative mRNA expression was calculated by getting the difference of the *ΔC*_*t*_ of the target gene from the group with no osteogenic induction (under maintenance medium) and under osteogenic induction (*ΔΔC*_*t*_). The relative value was expressed as RQ (2^−*ΔΔC*_*t*_).

### Alkaline Phosphatase and Von Kossa Staining

Mouse MSCs were cultured in 6-well plates at a density of 1.5 × 10^3^ /cm^2^, induced for osteogenesis by OIM and harvested at days 3, 5, 7 and 10. Samples were incubated for 1 hour with 500 μl of BCIP/NBT Liquid substrate system (Sigma) to examine Alkaline phosphatase (ALP) activity. Quantitative measurement of ALP activity was determined by fluorimetric method according to the manufacturer’s instruction (Abcam ab83371). Fluorescence intensity was measured at EX/EM 360/440 using a fluorescence microtiter plate reader (Tecan; Infinite M1000 PRO). For Von Kossa Staining, the cell monolayer was washed with PBS, fixed with 3.7% formaldehyde for 20 min then washed with distilled water. Subsequently, the cell monolayer was incubated with 1 ml of 2% silver nitrate (Sigma-Aldrich) at RT in the dark for 10 minutes followed by UV exposure for 45 minutes and washed with distilled water.

### Immunofluorescence Staining and Western Blot

For immunofluorescence staining, MSCs were grown in cover slips, washed with PBS, fixed with methanol, permeabilized with 0.1% Triton X- and blocked with 5% BSA in TBST. The samples were incubated with the following antibodies overnight at 4 °C: glucocorticoid receptor (D6H2L, 1:200, Cell Signaling), HDAC6, Runx2 (SC-5258 and SC-390715, 1:50 Santa Cruz). The appropriate secondary antibodies used are: Alexa Fluor 488-conjugated goat anti-rabbit, Alexa Fluor 586-conjugated goat anti-mouse, Alexa-fluor 688- conjugated donkey anti-goat (Life technologies) at a 1:250 dilution in PBS and counterstained with DAPI (1:2,000; Invitrogen Corporation, NY, USA). Images were acquired from at least 50 cells per treatment using Olympus Ax80 microscope. For co-localization detection, images were obtained using a confocal microscope (Zeiss LSM 700) and processed using LSM Image Browser (Zeiss).

For western blot, cells were harvested and lysed using RIPA buffer, and immunoblots were probed with glucocorticoid receptor (D6H2L, Cell Signaling) and Runx2 (SC-390715, Santa Cruz) antibodies.

### Sub cellular Fractionation

Cytoplasmic and nuclear protein fractions of MSCs were separated using ProteoExtract^®^ Subcellular Proteome Extraction Kit (Calbiochem) according to the manufacturer’s protocol. The purity of the fractions was determined by western blot using H3 antibody (1:1,000; Cell Signaling) as nuclear fraction control and Actin as cytosolic fraction control.

### Co-Immunoprecipitation

Mouse MSCs at day 0, 3 and 5 of osteogenic induction under 10^−7^ M dexamethasone were lysed with Pierce^TM^ IP lysis buffer (87787) with protease inhibitor cocktail. An estimated 350 μg of the pre-cleared lysate (total protein) was incubated with 1 μl of total GR antibody (D6H2L, Cell Signaling) or HDAC6 antibody (ab12173, Abcam), and incubated overnight at 4 °C under rotation. The complex was pulled down by 100 μl of Protein A- coupled separose beads (Millipore) Pierce^TM^ Protein A/G Agarose (20421) slurry for 2 hours at room temperature under rotation. The complex was centrifuged at 3000 rpm and the supernatant was discarded (unbound protein). The beads with the bound complex was washed 3x with 1 ml IP lysis buffer, centrifuged and the supernatant was discarded. Finally, the Ag-Ab complex was eluted from the beads by adding 50 μl of 2x SDS loading buffer and heated at 95 °C for 5 minutes. The samples, together with 20% input were analyzed by western blot.

### Chromatin Immunoprecipitation

MSCs were cross-linked with 1% formaldehyde, stopped with 250 mM Glycine, washed with TBS and lysed with 5 ml SDS buffer and the chromatin was sheared for 15 minutes at 40 Amp, 1 second on and 3 seconds off (Sonicator 4000, Misonix). Blocked protein A beads (Millipore) and pre-cleared lysates were incubated with 1 μg of either anti-glucocorticoid receptor-α (ab3580, Abcam), anti-Acetyl Histone H4 antibody (06–866, Millipore), HDAC6, Runx2 (SC-5258 and SC-390715 Santa Cruz), and IgG (Santa Cruz) at 4 °C overnight. The chromatin-protein-antibody complexes were pulled for 2 hours at 4 °C and washed in four successive steps with the following buffers as described previously^58^.

qPCR was performed using primers spanning the proximal and distal region of the osteocalcin promoter and a negative control for 3′ UTR and data was normalized with percent Input. The primers used for ChIP-qPCR and detailed method are listed in Supplementary Fig. S19.

### Inhibitor treatment

Tubacin inhibitor was purchased from Sigma (SML0065) and RU-486 (Sigma) was a gift from Prof. Jean-Cheng Kuo, Institute of Biochemistry, National Yang-Ming University, Taiwan.

### Statistical analysis

Statistical analysis was performed by one-way ANOVA with Tukey’s post hoc tests using Statistical Package for Social Science-12.0 software (SPSS Inc., Chicago, IL) or student’s t-test. The data were presented as mean ± SD of the results from two to three independent experiments. A p-value of < 0.05 was considered statistically significant.

## Additional Information

**How to cite this article**: Rimando, M. G. *et al*. Glucocorticoid receptor and Histone deacetylase 6 mediate the differential effect of dexamethasone during osteogenesis of mesenchymal stromal cells (MSCs). *Sci. Rep.* **6**, 37371; doi: 10.1038/srep37371 (2016).

**Publisher's note:** Springer Nature remains neutral with regard to jurisdictional claims in published maps and institutional affiliations.

## Supplementary Material

Supplementary Figures
