# Supplementary material for: Glucocorticoid receptor and Histone deacetylase 6 mediate the differential effect of dexamethasone during osteogenesis of mesenchymal stromal cells (MSCs)
Source: Sci Rep. 2016 Nov 30;6:37371. doi: 10.1038/srep37371 (PMC5128810; doi:10.1038/srep37371)
Supplement: Supplementary Figures [file srep37371-s1.pdf]

Supplementary Information

Glucocorticoid receptor and Histone Deacetylase 6 mediate the differential effect of dexamethasone during osteogenesis of Mesenchymal stromal cells (MSCs)

Marilyn G. Rimando, Hao-Hsiang Wu, Yu-An Liu, Chien-Wei Lee, Shu-Wen Kuo, Yin-Ping Lo, Kuo-Fung Tseng, Yi-Shiuan Liu, Oscar Kuang-Sheng Lee

Supplementary Table 1. Primer sequences used for Real Time Reverse Transcription and Polymerase Chain Reaction. Intron spanning primers were designed from Universal Probe Library (Roche Applied Sciences, Mannheim, Germany) for real time PCR using TaqMan universal probe system.

Supplementary Figure 1. Changes in cellular morphology and Alkaline Phosphatase (ALP) activity during osteogenic differentiation of mouse mesenchymal stromal cells (mMSCs). mMSCs were treated with osteogenic induction medium (OIM) consisting of high glucose DMEM supplemented with 0.2 mM Ascorbic Acid, 10 mM β-glycerophosphate at various Dex concentrations: 0M, 10^{-7}M, and 10^{-5}M. Samples were collected at various time points : d3, d5, d7, d10 and were stained with BCIP/NBT Liquid substrate system (Sigma) and incubated for 1 hour to examine Alkaline phosphatase activity. Alkaline phosphatase expression was observed as early as day 3 in differentiating MSCs and increases expression at day 5 and 7 at increasing dexamethasone dose. Scale bar= 100 µm

Supplementary Figure 2. Changes in cellular morphology during osteogenic differentiation of human mesenchymal stromal cells (hMSCs). hMSCs were treated with osteogenic medium (OIM) consisting of IMDM supplemented with 0.2 mM ascorbic acid and 10 mM β-glycerol phosphate at various Dex concentrations: 0M, 10^{-7}M, and 10^{-5}M. OIM was replaced every 3 days and Samples were collected at various time points: day 7, day 14, day 21. As osteoblast differentiation progress, MSCs exhibit a broader and flattened polygonal morphology. Scale bar= 100 µm

Supplementary Figure 3. Increasing concentration of Dex did not affect the protein level of RUNX2 during osteogenic differentiation of mMSC. MSCs were cultured under maintenance (low-glucose; LG DMEM for mouse and IMDM for human) and osteogenic induction medium with various Dex concentrations (0M, 10^{-9}, 10^{-8}, 10^{-7}, 10^{-6}, 10^{-5} M). Cell lysates were collected at days 0, 3, 5 and 7 of osteogenic induction from mouse MSC and at days 7 and 14 in humans. Samples were immunoblotted against RUNX2 and GAPDH as loading control.

Supplementary Figure 4. Expression of total glucocorticoid receptor in mMSCs, hMSCs and mature human fetal osteoblast (hFOB). MSCs were cultured in Low glucose (LG) DMEM and hFOB in Iscove's modified Dulbecco's medium (IMDM) supplemented with 10% fetal bovine serum and 1x PSG. Samples were stained with total GR antibody at day 3 and day 14 of mMSCs and hMSCs respectively and after 24 hours culture for hFOB, and counterstained with DAPI. Images were acquired using a fluorescence microscope (Olympus Ax80).

Supplementary Figure 5. Protein expression of glucocorticoid receptor (GR) in hMSCs in the absence and presence of dexamethasone. MSCs were cultured in osteogenic induction medium under 0M, 10^{-7} M and 10^{-5}M Dex concentrations. Samples were stained with total GR antibody at day 14 of differentiation and counterstained with DAPI. The GR is predominantly found in the cytoplasm and translocated into the nucleus in the presence of Dex. High Dex concentration slightly decreased GR expression. Images were acquired using a fluorescence microscope (Olympus Ax80).

Supplementary Figure 6. Protein expression of total glucocorticoid receptor (GR) in human fetal osteoblast (hFOB) in the presence of dexamethasone. hFOB were cultured in Iscove's modified Dulbecco's medium (IMDM) supplemented with 10% fetal bovine serum and 1x PSG. The GR response upon treatment with dexamethasone is similar to differentiating mouse and human MSC. GR is predominantly localized in the cytoplasm in the absence of Dex and translocates to nucleus in the presence of 10^{-7} and 10^{-5}M Dex. At high Dex concentration (10^{-5}M), the nuclear translocation signal of GR is weaker compared to 10^{-7} Dex concentration. Samples were imaged under Olympus Ax80 fluorescence microscope. Scale bar= 20 µm.

Supplementary Figure 7. Subcellular fractionation of GR. Subcellular fractionation of GR during osteogenic differentiation of mouse MSCs using ProteoExtract®Subcellular Proteome Extraction Kit (Calbiochem). F1- cytoplasmic fraction; F2- membrane organelles fraction; F3- nuclear fraction; LG- low glucose maintenance medium.

Supplementary Figure 8. HDAC6 is expressed in mMSCs at day 7 of osteogenic induction under varying concentrations of dexamethasone (Dex): 0M, 10^{-7}M and 10^{-5}M. HDAC6 co-localized with GR in the cytoplasm in the absence (0M) and presence (10^{-7}M) of Dex as marked by the appearance of colocalization puncta (yellow/pink). Samples were imaged under confocal microscope (Zeiss LSM 700) and processed using LSM Image Browser (Zeiss). Scale bar= 20µm.

Supplementary Figure 9. A. Schematic representation of the proximal and distal region of the Osteocalcin promoter. B. Primer sequences used for the CHIP assay.

Supplementary Figure 10. HDAC6 expression upon inhibition of GR activity by RU-486 (2 µM) at day 3 of osteogenic induction with 10^{-7} M Dex concentration. Cells were stained with total GR and HDAC6 with Alexa Fluor 488-conjugated goat anti-rabbit and Alexa Fluor 586-conjugated goat anti-mouse secondary antibodies respectively, counterstained with DAPI, imaged under Zeiss LSM 700 confocal microscope and processed using LSM Image Browser (Zeiss). Scale bar= 20 µm.

Supplementary Figure 11. Glucocorticoid receptor forms a complex with Runx2. Co-immunoprecipitation of Glucocorticoid receptor (GR) with Runx2 in mMSCs and hFOB under day 3 of osteogenic differentiation and 24 hours treatment with 0, 10^{-7}M and 10^{-5}M concentrations of Dex respectively.

Supplementary Figure 12. Full-length SDS-PAGE blots of Figure 3a and 3b. a-b. Protein expression of GR was detected from MSCs under maintenance medium (LG) and osteogenic induction medium (OIM) for 3 and 7 days (D3, D7) with 0M, 10^{-9} to 10^{-5}M Dex and from mature bone cells: Human Fetal Osteoblast (hFOB) and osteosarcoma cell line (SaOS-2). c-d. Cyclohexamide (CHX) treatment in undifferentiated mMSCs maintained in low glucose (LG) DMEM and differentiating MSCs at day 5 in OIM. De-novo GR protein synthesis was inhibited by 75 µg/ml cyclohexamide (CHX) for 6 hours and 8 hours treated in the presence or absence of 10^{-7} and 10^{-5} M Dex. Blots were derived from the same experiments and were processed in parallel.

Supplementary Figure 13. Full-length SDS-PAGE blot of Figure 5d. Complex formation between GR and HDAC6 (a,b) by Co-immunoprecipitation from mouse MSCs at days 0, 3,5 (D0, D3, D5) of osteogenic induction with 10^{-7}M Dex. IP: immunoprecipitation; UF: supernatant (unbound fraction) of IP pulldown.

Supplementary Information Table 1.

Primer sequences used for Real Time Reverse Transcription

Gene	***Abbreviation***	***Sequence (mouse)***	***Sequence (human)***
Runt-Related Transcription Factor 2	*Runx2 /Cbfa-1*	F- gcccaggcgtatttcaga<br>R- tgcctggctcttcttactgag	F - gtgcctaggcgcatttca<br>R- gctcttcttactgagagtggaagg
Alkaline Phosphatase	*ALP*	F- ttaagggccagctacaccac<br>R-agggacctgagcgttggt	F- agaaccccaaaggcttcttc<br>R- cttggcttttccttcatggt
Osterix	*OSX/SP7*	F- tgcttcccaatcctatttgc<br>R- agctcaggggggaatcgag	F- catctgcctggctccttg<br>R- caggggactggagccata
Osteopontin	*OPN*	F- ggaggaaaccagccaagg<br>R- tgccagaatcagtcactttcac	F- gagggcttggttgtcagc<br>R- caattctcatggtagtgagttttcc
Collagen type 1	*Col1a1*	F- agacatgttcagctttgtggac<br>R- gcagctgacttcagggatg	F- cccctggaaagaatggagat<br>R- aatcctcgagcaccctgag
Osteocalcin/ Bglap1	*OCN*	F- agactccggcgctacctt<br>R- ctcgtcacaagcagggttaag	F- tgagagccctcacactcctc<br>R- acctttgctggactctgcac
Bone Sialoprotein	*BSP*	F- gaaaatggagacggcgatag<br>R- cattgttttcctcttcgtttga	F- caatctgtgccactcactgc<br>R- cagtcttcattttggtgattgc
Nuclear receptor subfamily 3/ Glucocorticoid Receptor	*NR3C1*	F- caaagattgcaggtatcctatgaa<br>R- tggctcttcagaccttcctt	F- ccttctgcgttcacaagcta<br>R- ttctttggagtccatcagtgaat
Histone Deacetylase 6	*HDAC6*	F- ggctgagattcggaatgg<br>R- cccatccataagattgtgctg	F- agttcaccttcgaccaggac<br>R- gccagaacctaccctgctc
40S ribosomal protein S18	*RPS18*	F- cttccacaggaggcctacac<br>R- tggtgttgagtactcgcaaaat	
Glyceraldehyde 3-phosphate dehydrogenase	*GAPDH*	F- agcttgtcatcaacgggaag<br>R- tttgatgttagtggggtctcg	F- agccacatcgctcagacac<br>R- gcccaatacgaccaaatcc

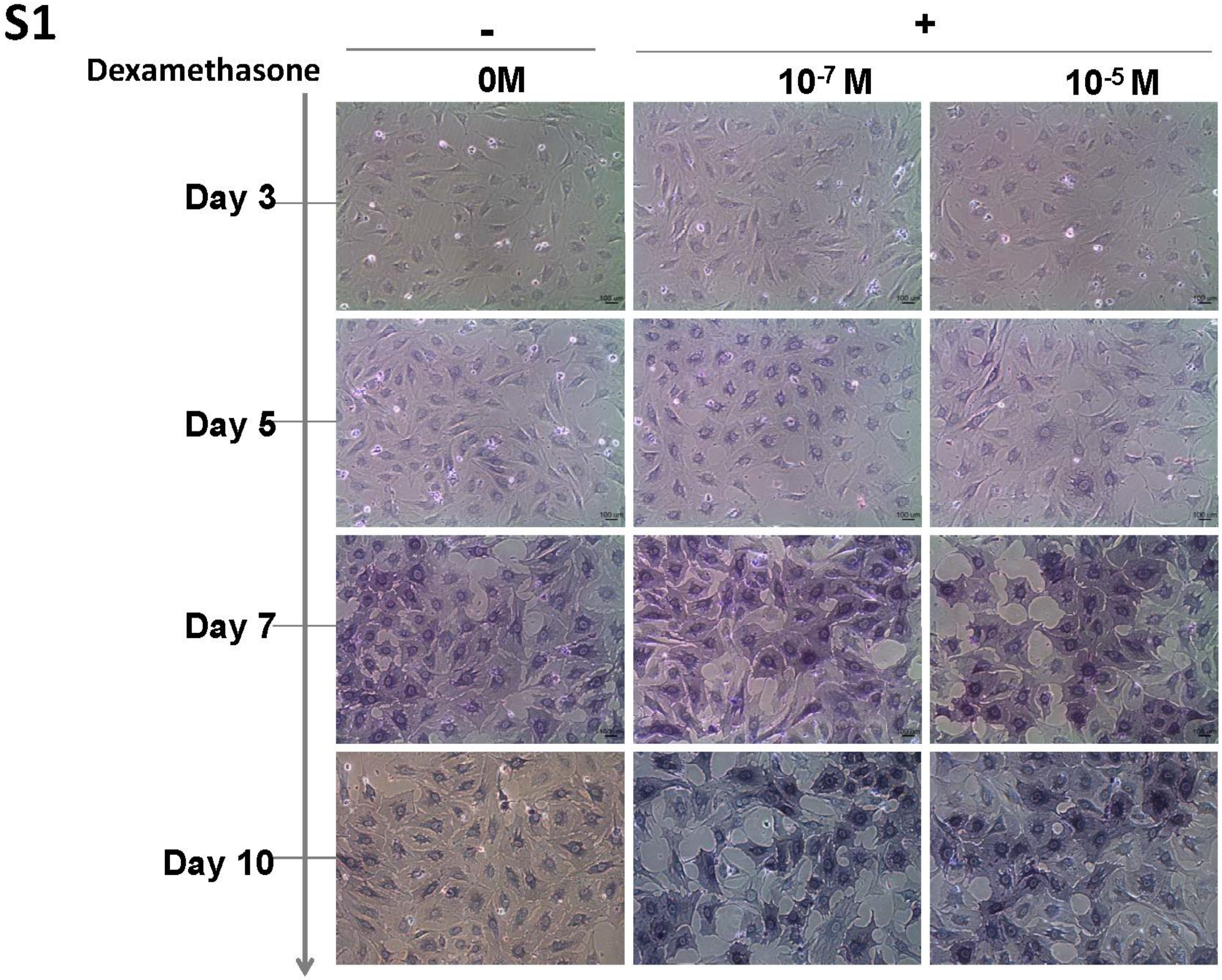
S1
Dexamethasone
-
+
0M
10-7 M
10-5 M
Day 3
Day 5
Day 7
Day 10

S2

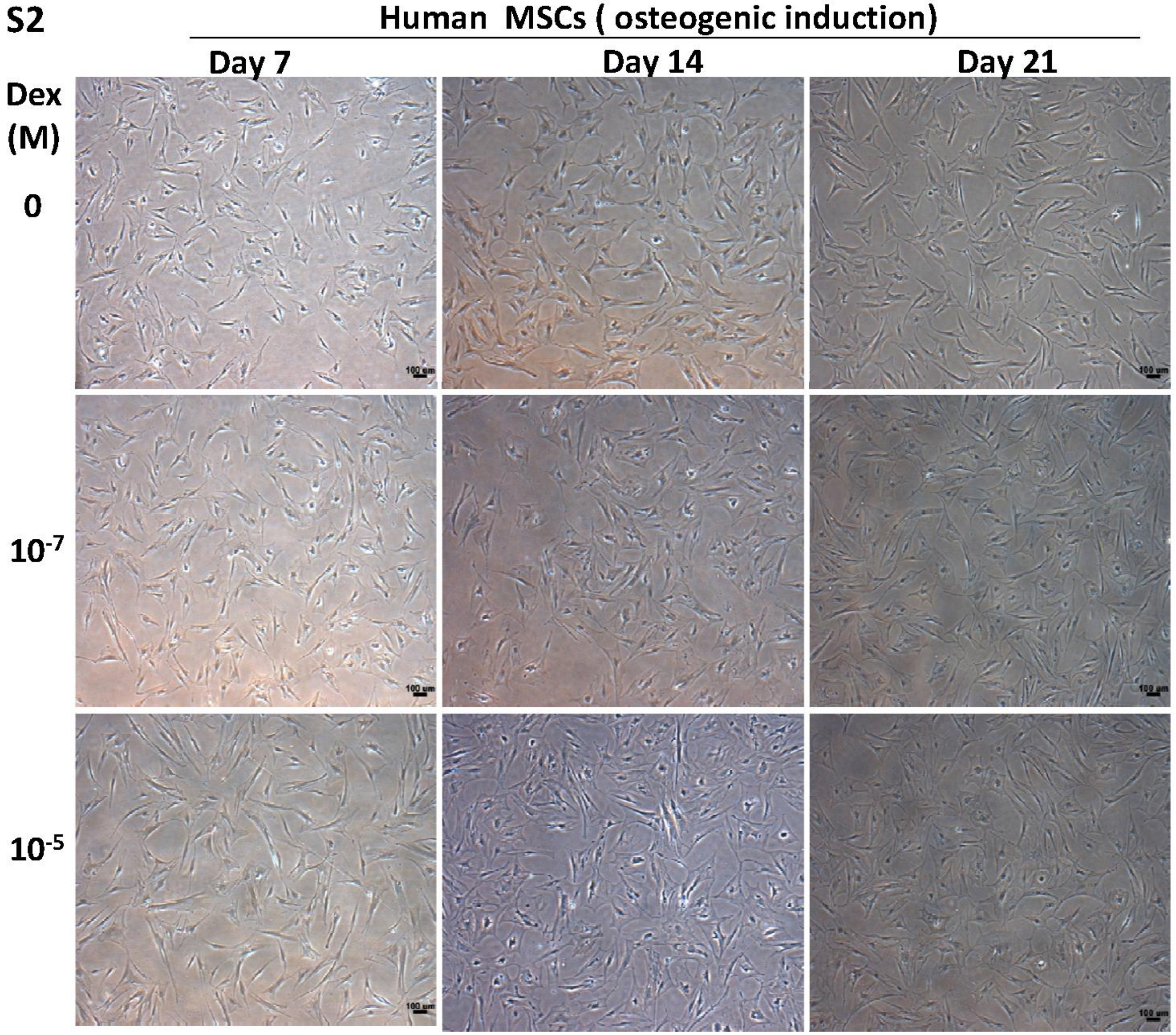

Human MSCs (osteogenic induction)
Day 7
Day 14
Day 21
Dex (M)
0
10^{-7}
10^{-5}
100 um

S3

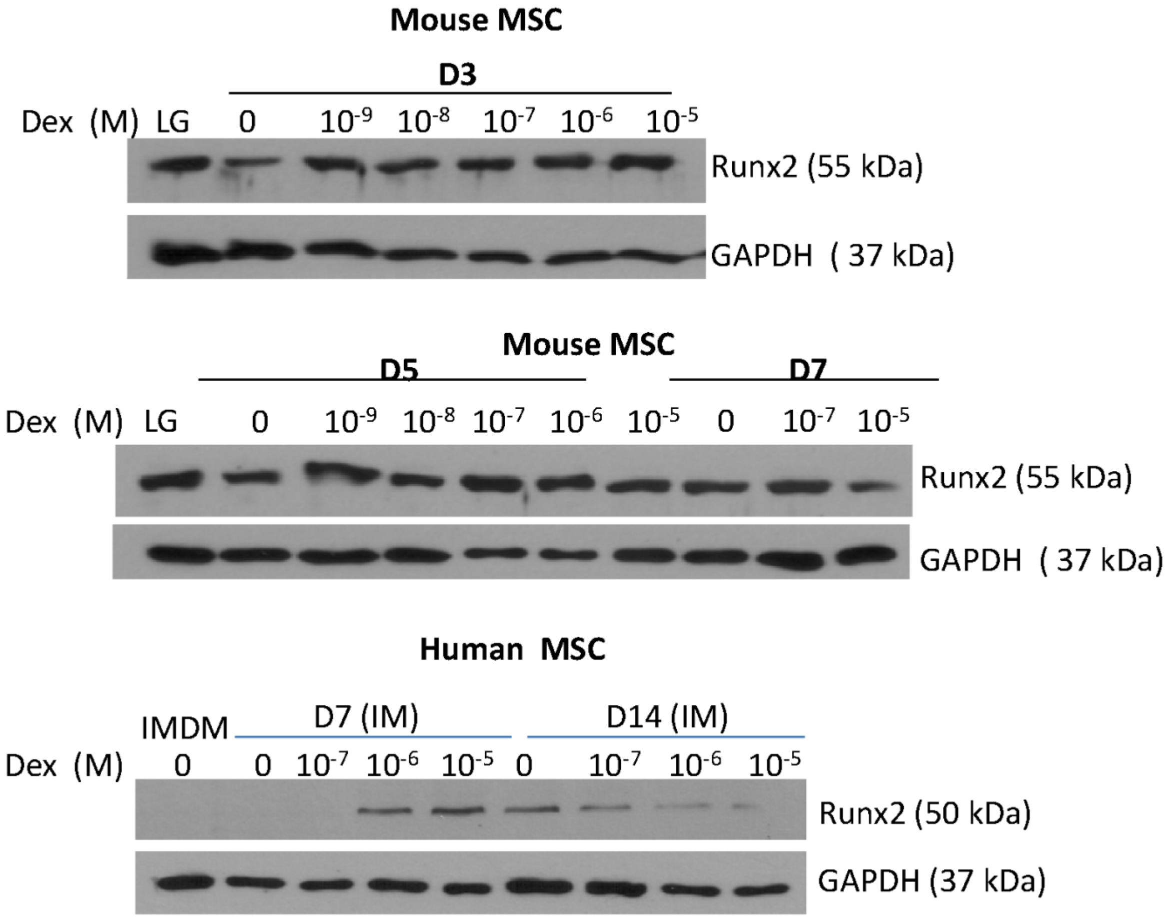
Mouse MSC
D3
Dex (M) LG 0 10^-9 10^-8 10^-7 10^-6 10^-5
Runx2 (55 kDa)
GAPDH (37 kDa)
Mouse MSC
D5
D7
Dex (M) LG 0 10^-9 10^-8 10^-7 10^-6 10^-5 0 10^-7 10^-5
Runx2 (55 kDa)
GAPDH (37 kDa)
Human MSC
IMDM
D7 (IM)
D14 (IM)
Dex (M) 0 0 10^-7 10^-6 10^-5 0 10^-7 10^-6 10^-5
Runx2 (50 kDa)
GAPDH (37 kDa)

S4

total glucocorticoid receptor

mMSC (Day 0) **hMSC (Day 14)** **hFOB (48 hours)**

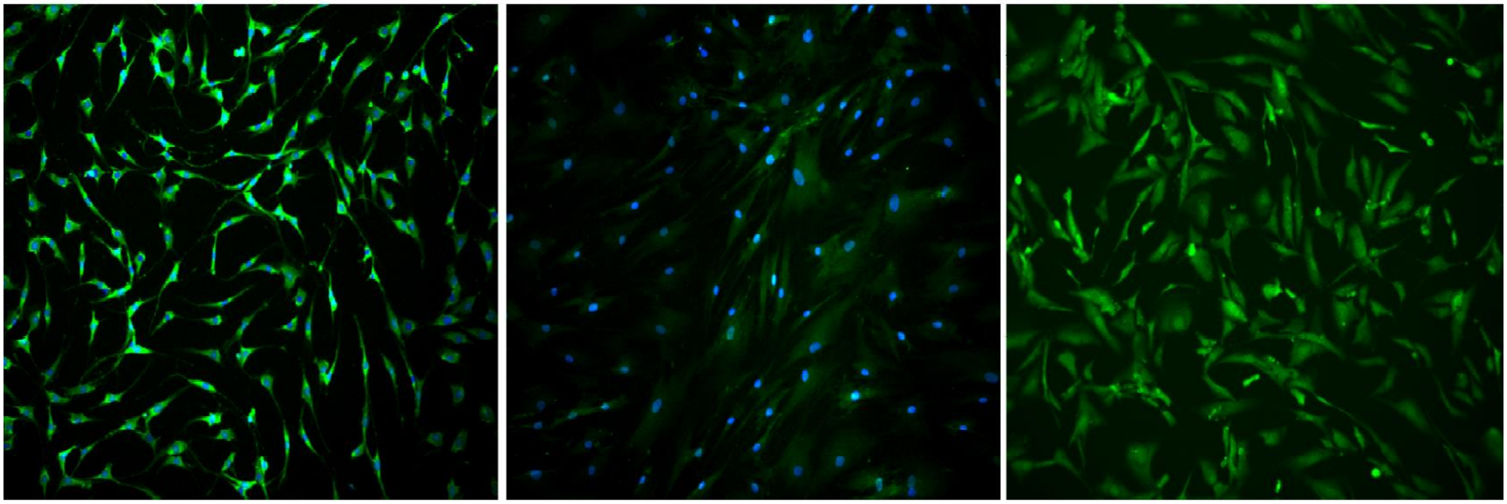

S5

Human MSCs (day 14)

Dex **OM** **10^{-7}M** **10^{-5}M**

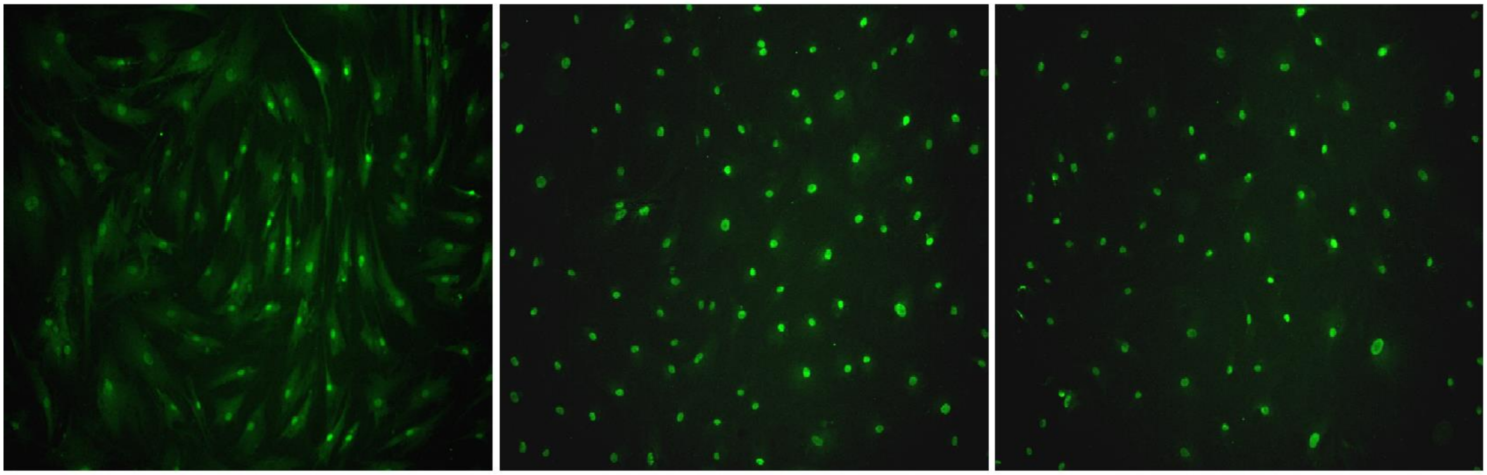

S6

Human Fetal Osteoblast (hFOB) 48 hours

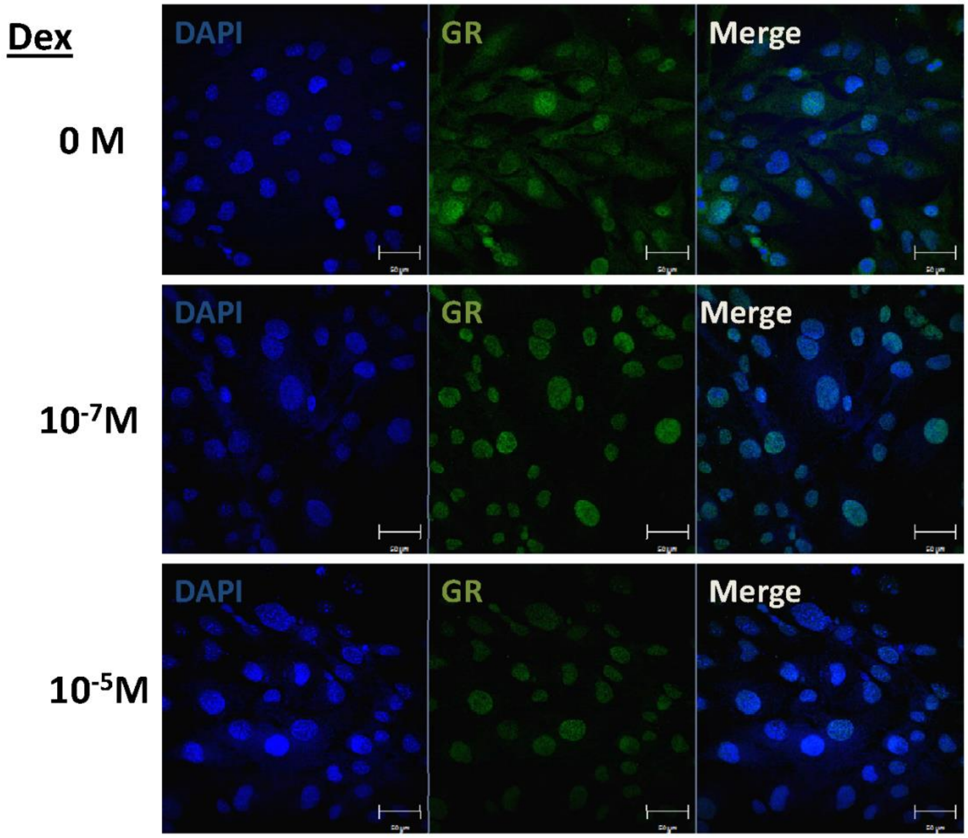

S7

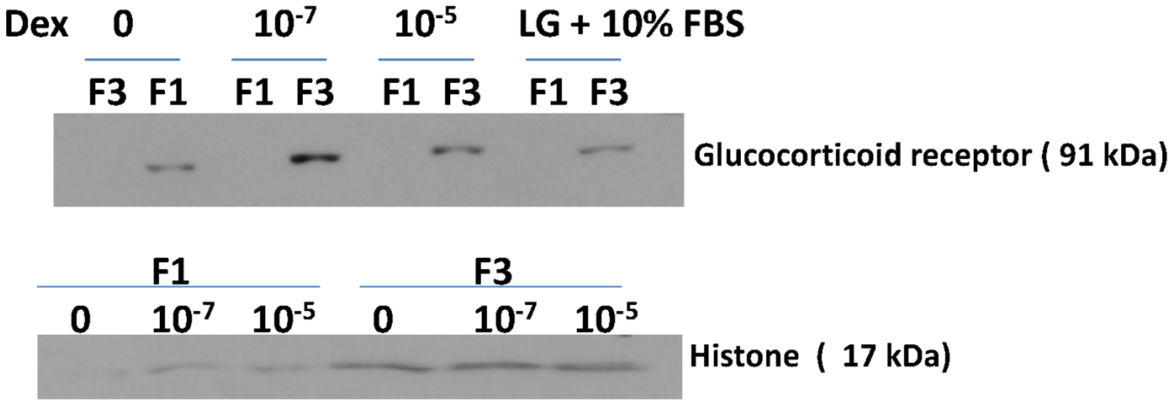
Dex 0 10-7 10-5 LG + 10% FBS
F3 F1 F1 F3 F1 F3 F1 F3
Glucocorticoid receptor (91 kDa)
F1 F3
0 10-7 10-5 0 10-7 10-5
Histone (17 kDa)

S8

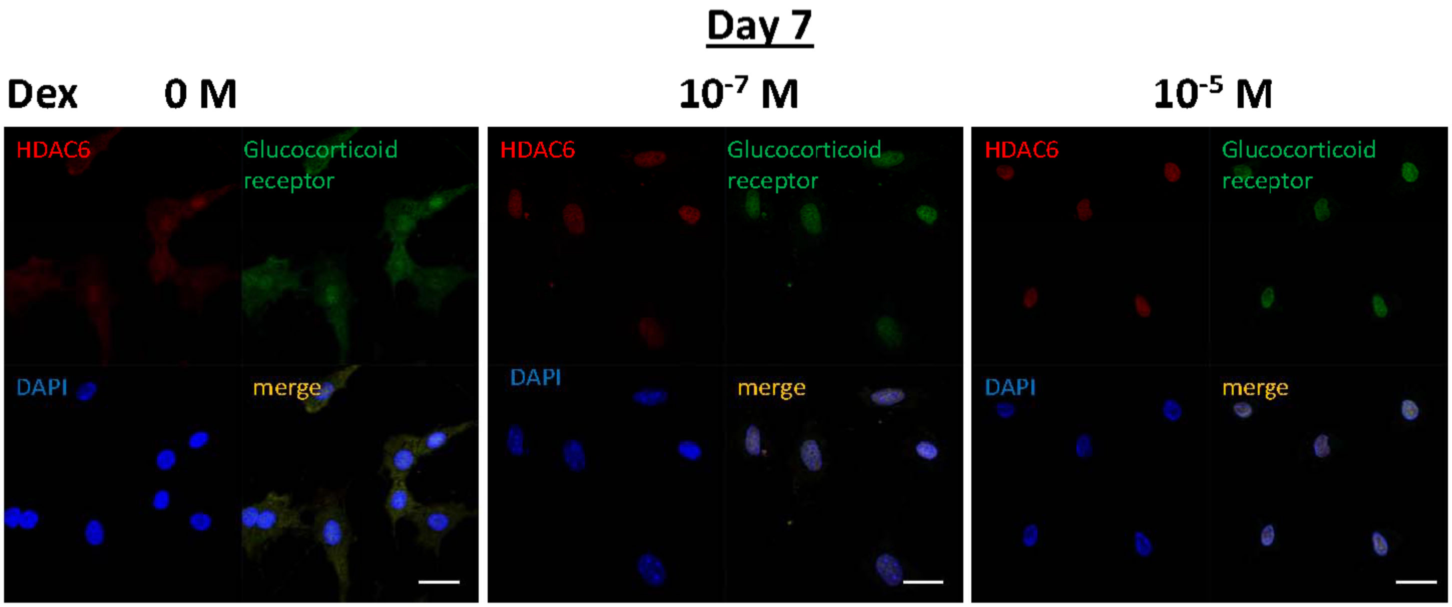
Day 7
Dex 0 M
10-7 M
10-5 M
HDAC6
Glucocorticoid receptor
DAPI
merge

S9

a

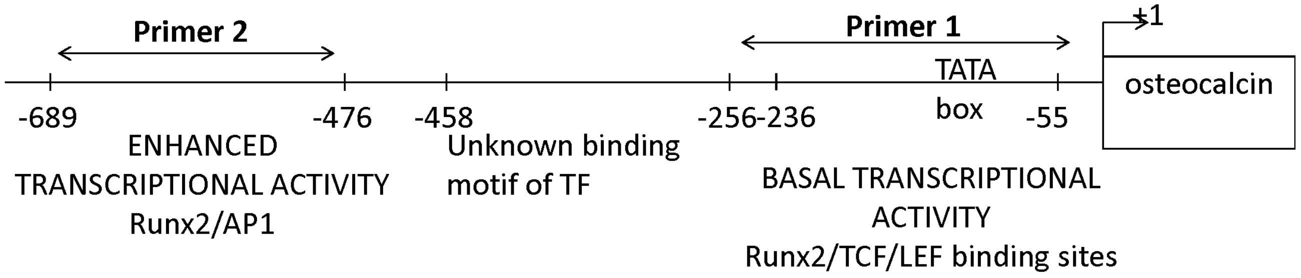

b

Primer 1	5′-GCC CCT CAG GGA AGA GGT CTT-3′ and 5′-CTG CAC CCT CCA GCG TCC AG-3	Bone 2009 Sep; 45(3):579-89
Primer 2	5′-CGC TCT CAG GGG CAG ACA CT and 5′-GCA CCC TCC AGC ATC CAG TA	Mol Endocrinol. 2006 Oct;20(10):2432-43
Primer 3	3UTR primer<br>5′-GAT CCC ATA TCA GCC AGC AC-3′<br>5′-GAC TGC CCT GGA TCA CAA GT-3′	Bone 2009 Sep;45(3):579-89

S10

Day 3

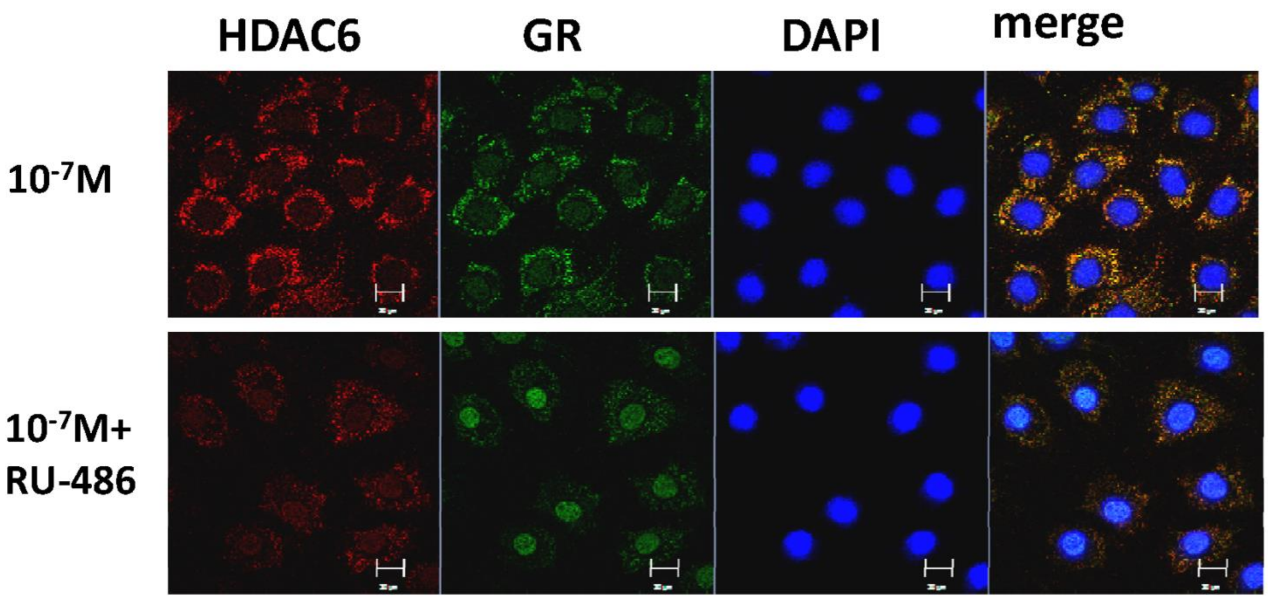

S11

a

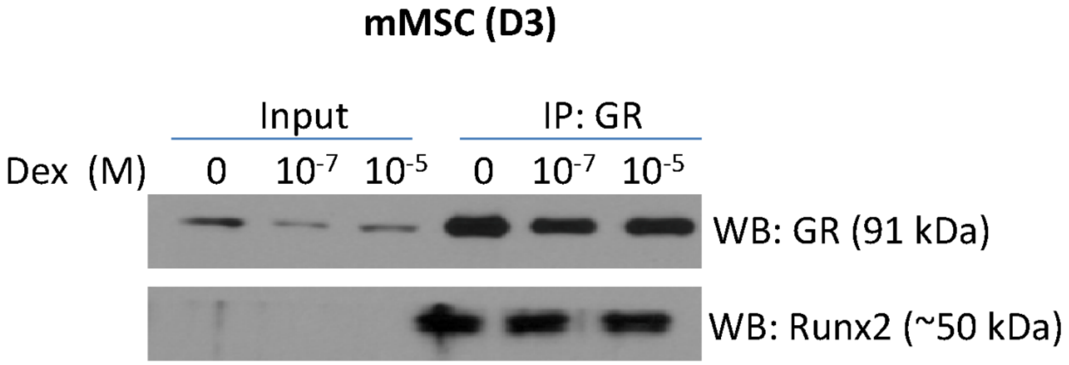
mMSC (D3)
Input
IP: GR
Dex (M)
0
10-7
10-5
0
10-7
10-5
WB: GR (91 kDa)
WB: Runx2 (~50 kDa)

b

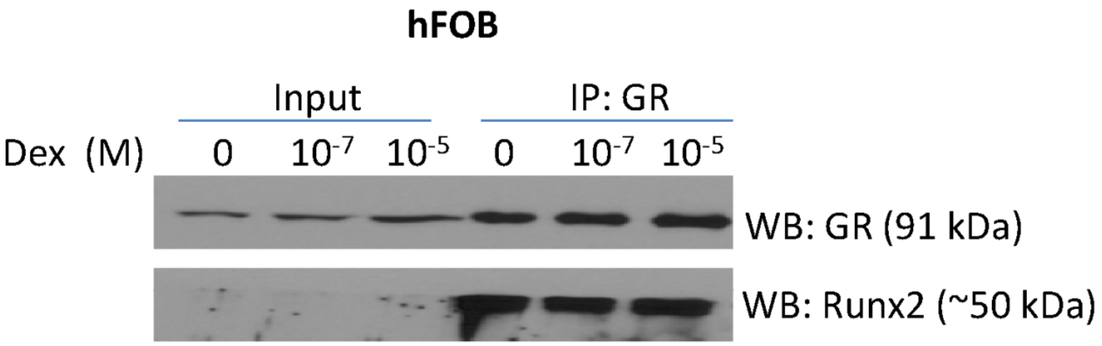
hFOB
Input
IP: GR
Dex (M)
0
10-7
10-5
0
10-7
10-5
WB: GR (91 kDa)
WB: Runx2 (~50 kDa)

S12

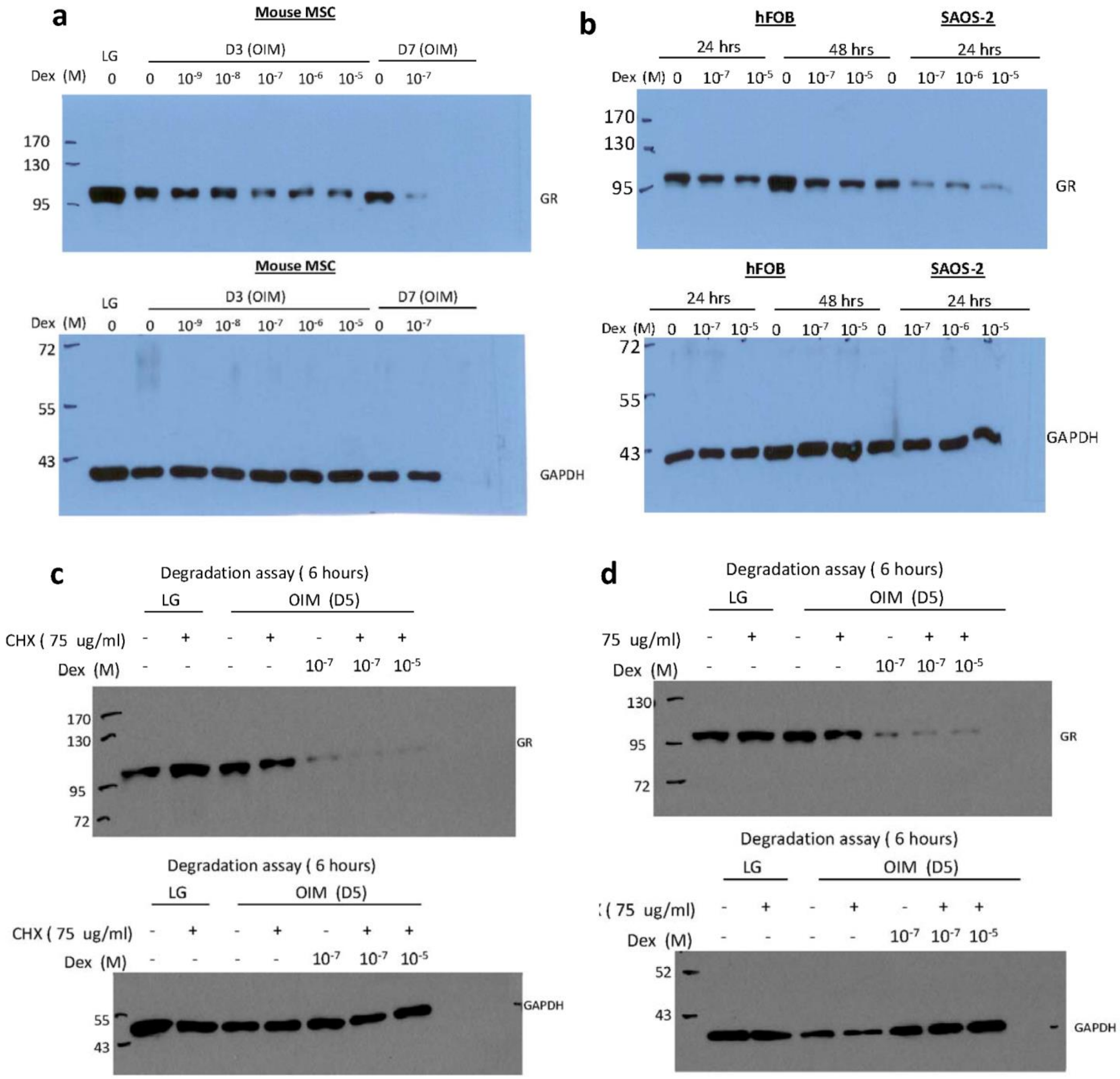
a
Mouse MSC
LG
D3 (OIM)
D7 (OIM)
Dex (M) 0 0 10-9 10-8 10-7 10-6 10-5 0 10-7
170
130
95
GR
Mouse MSC
LG
D3 (OIM)
D7 (OIM)
Dex (M) 0 0 10-9 10-8 10-7 10-6 10-5 0 10-7
72
55
43
GAPDH
b
hFOB
SAOS-2
24 hrs
48 hrs
24 hrs
Dex (M) 0 10-7 10-5 0 10-7 10-5 0 10-7 10-6 10-5
170
130
95
GR
hFOB
SAOS-2
24 hrs
48 hrs
24 hrs
Dex (M) 0 10-7 10-5 0 10-7 10-5 0 10-7 10-6 10-5
72
55
43
GAPDH
c
Degradation assay (6 hours)
LG
OIM (D5)
CHX (75 ug/ml) - + - + - + +
Dex (M) - - - - 10-7 10-7 10-5
170
130
95
72
GR
Degradation assay (6 hours)
LG
OIM (D5)
CHX (75 ug/ml) - + - + - + +
Dex (M) - - - - 10-7 10-7 10-5
55
43
GAPDH
d
Degradation assay (6 hours)
LG
OIM (D5)
75 ug/ml) - + - + - + +
Dex (M) - - - - 10-7 10-7 10-5
130
95
72
GR
Degradation assay (6 hours)
LG
OIM (D5)
(75 ug/ml) - + - + - + +
Dex (M) - - - - 10-7 10-7 10-5
52
43
GAPDH

S13

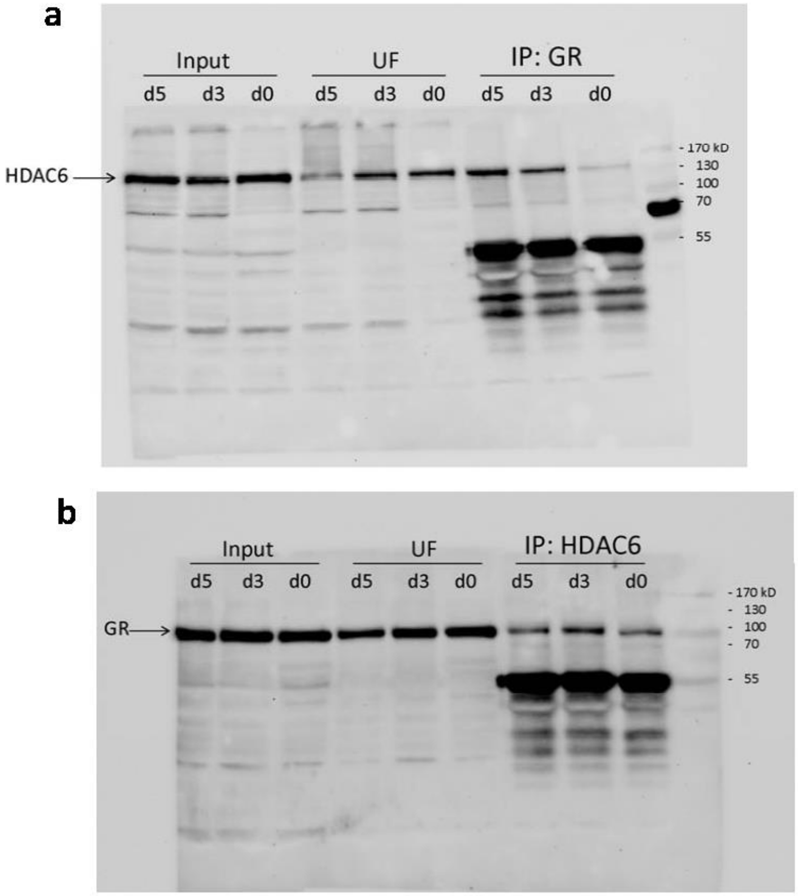
a
Input
UF
IP: GR
d5
d3
d0
d5
d3
d0
d5
d3
d0
HDAC6
- 170 kD
- 130
- 100
- 70
- 55
b
Input
UF
IP: HDAC6
d5
d3
d0
d5
d3
d0
d5
d3
d0
GR
- 170 kD
- 130
- 100
- 70
- 55
